# Early life stress and pubertal predictors of subsequent substance use in a national diverse sample of adolescents: Sex and substance type matter

**DOI:** 10.1016/j.drugalcdep.2025.112551

**Published:** 2025-01-13

**Authors:** Alexandra Donovan, Shervin Assari, Christine Grella, Magda Shaheen, Linda Richter, Theodore C. Friedman

**Affiliations:** a Department of Internal Medicine, College of Medicine, Charles R. Drew University of Medicine and Science, 1731 E. 120th St, Los Angeles, CA 90059, USA; b Department of Family Medicine, College of Medicine, Charles R. Drew University of Medicine and Science, 1731 E. 120th St, Los Angeles, CA 90059, USA; c Integrated Substance Abuse Programs, Department of Psychiatry and Biobehavioral Sciences, University of California, 10911 Weyburn Ave, Suite 200, Los Angeles, CA 90024-2886, USA; d Partnership to End Addiction, 711 Third Ave, 5th Floor, Suite 500, New York City, NY 10017, USA

**Keywords:** Sex, Adolescence, Substance use, Stress, Adversity

## Abstract

**Background:**

Early life stress (ELS) increases the risk of substance use disorder (SUD) in adulthood. The pathway from ELS to SUD is hypothesized to be influenced by sex. We examine the impact of ELS on adolescent first substance use, a common precursor to adult SUDs, and test for sex differences in the relationship between ELS and risk of first use of alcohol, nicotine, and marijuana.

**Methods:**

Individuals from the Adolescent Brain Cognitive Development study (ABCD; N = 8608 US children aged 9–10 at baseline) were assessed for multiple measures of ELS (Environmental, Family, Trauma), covariates (race/ethnicity, age in months at baseline, pubertal development score), and first substance use. Cox proportional hazards regression was performed for total population and stratified by sex, generating adjusted hazard ratios (aHRs) of first substance use.

**Results:**

One unit increase in ELS Total scores significantly increased aHRs of first use of all substances. Dimensional ELS analysis revealed associations with risk that differed across sex, type of ELS, and substance. Females with higher ELS Environmental scores display a significant increase in risk of first use across all substances, while males with higher ELS Trauma scores showed a significant increase in risk of first nicotine and marijuana use.

**Conclusions:**

Findings highlight sex differences in the association of ELS Environmental and Trauma scores with the risk of first substance use, while illustrating the consistency of the association of ELS Family scores with risk. Existing family-based prevention and intervention strategies should consider addressing sex differences in adolescent substance use risk and protection.

## 1. Introduction

Early life stress (ELS) is a construct comprising adverse childhood experiences of maltreatment and family conflict, as well as additional societal stressors such as low socioeconomic status (SES) or neighborhood violence (Donovan et al., 2024). Though much work has been done examining the effects of adverse childhood experiences (Felitti et al., 1998), studies have identified a need to place these adverse events in the broader context of SES and neighborhood stressors in order to identify strategies for intervention (Fagan et al., 2015). ELS has been linked to an increased risk of adult metabolic and cardiovascular disease, mental health disorders, and substance use disorders (SUD) (Barnhart et al., 2022; Felitti et al., 1998). More than 20 % of adolescents in the United States have experienced ELS at some point, affecting adolescent health behaviors (Hoffman et al., 2019). ELS is associated with earlier substance use initiation, and studies have shown that the age of substance use initiation is inversely related to the number of ELS experienced (Rothman et al., 2008). Adolescent substance misuse is especially troubling, as younger age at initiation is correlated with increased odds and severity of SUD (King and Chassin, 2007). The mechanism by which ELS influences these health outcomes is not entirely known, but research indicates early life involvement of genetic, epigenetic, cultural, social, and psychological effects on health and health behaviors (Donovan et al., 2024).

One potential pathway connecting ELS to adult health outcomes occurs during adolescence. The overlapping processes of puberty, brain maturation, and physical development that define adolescence require plasticity that leaves the door open for ELS-induced neuroendocrine and psychological adaptions. ELS is associated with altered timing (Herting et al., 2020) and tempo of puberty (Hamlat et al., 2022), which have both been associated with an increase in early substance use (Castellanos-Ryan et al., 2013; Downing and Bellis, 2009) and psychopathology (Niu et al., 2023). Additionally, researchers observed sex differences in sensitivity to ELS-related changes in hormone levels and pubertal maturation processes (Phan et al., 2021), with females showing hormonal coupling sensitivity to ELS. However, pubertal timing is also affected by race and ethnicity with Black, Native American/Alaska Native and Hispanic females undergoing puberty earlier than Asian or White females (Senger-Carpenter et al., 2024). Thus, it is important to consider the layered effects of sex, age, race, and ethnicity when examining associations between ELS and puberty.

These shifts in neuroendocrine systems alter the development of limbic, reward, and executive control areas of the brain (Ladouceur et al., 2019) implicated in internalizing disorders such as depression or anxiety. Prior to puberty, these disorders were equally prevalent among females and males; it is not until puberty begins to shape the brain that internalizing disorders become more prevalent in females. Early puberty is strongly associated with an increase in both internalizing and externalizing disorders in females (Niu et al., 2023). These internalizing disorders may in turn lead to initiation of substance use as a coping mechanism (Marceau and Abel, 2018) to escape negative emotional states (Heitzeg et al., 2018) or “forget bad memories” (Connolly, 2024). Thus, the ELS-associated pubertal timing effects may be stronger among females, leading to the initiation of substance use.

Sex and gender differences in the impact of ELS on substance use behaviors have been observed in both humans and animal models (Winter et al., 2023). For example, adult and adolescent studies have found that females who experienced the highest rate of ELS were significantly more likely to misuse substances than their male counterparts (Evans et al., 2017; Zaidi et al., 2022). This suggests that adolescent females may be more sensitive to ELS than males and will display a stronger relationship between ELS and substance use initiation relative to males. The current study investigates this hypothesis by testing for associations between ELS score and first use of a substance across male and female adolescents in the Adolescent Brain Cognitive Development (ABCD) study, aiming to disentangle the effects of ELS, puberty, and sex on subsequent substance use initiation.

## 2. Methods

### 2.1. Study design

This longitudinal study used data from baseline through the first 3 waves of the Adolescent Cognitive Development (ABCD) study. Recruiting 11,868 youth ages 9–10 across 22 research sites, the ABCD study collected data from a diverse sample of youth and parents in yearly in-person visits and midyear phone interviews (Barch et al., 2018; Garavan et al., 2018; Lisdahl et al., 2018; Volkow et al., 2018). All ABCD study procedures were approved by the Institutional Review Board centralized at University of California, San Diego. Informed consent was obtained from parents/guardians, and assent from youth during each session. The current study used ABCD Data Release 5.1, which contained data from the full population at baseline (ages 9–10) through Year 3 (ages 12–13). At baseline, age in months, sex as assigned at birth (male, female), and race/ethnicity were reported by parents/guardians. Race/ethnicity is a covariate and is represented as a 5-level category: Asian, Black, Hispanic, Other (Native American/Alaska Native, Multiracial Non-Hispanic), or White (Martz et al., 2022).

### 2.2. ELS score

Baseline measures were used to generate a total ELS score (ranging from 0 to 50) and separate scores in each of three dimensions: Environmental (scores 0–11), Family (scores 0–22), and Trauma (scores 0–17). Total ELS score was used to capture the interrelatedness of the dimensions explored and represent the cumulative impact of lower-level ELS across dimensions. This cumulative impact is important as each dimension can affect another, i.e. belonging to a low-income family (Environmental) can cause strain on familial relationships (Family) (Leung et al., 2020). Measures included in each ELS dimension were derived from previous ABCD studies (Brieant et al., 2023; Hoffman et al., 2019; Orendain et al., 2023) and literature linking ELS with substance use (Evans et al., 2017; Rothman et al., 2008). Measures described stressors present within the past 12 months, except for family history and ELS Trauma measures, which described lifetime history. Participants missing values for measures used to calculate ELS scores were removed (N = 1207).

ELS Total score was separated into Environmental, Family, and Trauma scores. ELS Environmental score reported on parental education (1 point for GED, high school diploma, or incomplete high school), household status (1 point if divorced, widowed, or separated), financial sufficiency (1 point each for inability to pay rent, food, medical or dental care, or utilities in the previous 12 months, up to 7 points), and linked external data measuring low neighborhood income (Area Deprivation Index, 1 point if median neighborhood income < $50,000). ELS Family score includes measures of conflict in family environment (FES, parent and youth report averaged, up to 9 points), level of emotional support provided by caregiver (CRPBI, youth report reverse scored, up to 2 points), and family history of psychopathology including substance use (FHX, parent report, up to 11 points). ELS Trauma score draws from the KSADS-PTSD module parent report, assigning 1 point for each traumatic event experienced (such as child maltreatment, witnessing or experiencing violence, or natural disasters, see Appendix A) up to 17 points.

### 2.3. Pubertal status

Parental report of pubertal development utilizing the Pubertal Development Scale (PDS, referred to as “puberty”) at baseline was used to avoid over- or under- reporting of status that can occur with youth reports (Cheng et al., 2021; Herting et al., 2020). Parents indicated to what degree characteristics such as facial hair or breast development were present in their child (1 = “has not yet started”, 2 = “has barely started”, 3 = “is definitely underway”, and 4 = “seems complete”). Scores were averaged across all questions answered for both males and females. Though not as precise as the gold standard Tanner staging, the ease of administration and versatility are distinct advantages in large datasets such as the ABCD study, and reports of PDS have been found to correlate better with hormonal measures than clinician-assessed Tanner staging (Cheng et al., 2021). The baseline value was used for all calculations, as independent variables were assessed cross-sectionally.

### 2.4. Substance use measures

Measures were calculated using data from baseline through year 3 of the ABCD study. Time was converted into months, where baseline interview = 0 months. Subjects were removed if no substance use data were available after baseline (N = 123). Based on prior studies (Sullivan et al., 2022) first use of a substance was defined as consumption of a full standard unit of alcohol or greater than a puff or taste of nicotine or marijuana. Time to first substance use was recorded as the number of months from baseline to the interview in which the participant reported use and ranged from 6 months to 36 months in the analytical sample. Respondents reporting use of alcohol, nicotine, or marijuana at baseline were removed from the analysis (N = 70) so that first use reflected initiation after baseline. Use did not include any religious or ceremonial consumption of alcohol or tobacco as identified by questions “Some people have alcohol as part of a religious ceremony such as in church or at a Seder dinner. Have you had a drink of alcohol that was NOT part of a religious service?” and “Some people smoke as part of a religious ceremony. Have you ever smoked a full tobacco cigarette, cigar, or pipe, or had multiple puffs of hookah or an electronic cigarette that was NOT part of a religious ceremony?”.

### 2.5. Statistical analysis

SPSS Version 27.0 was used to perform all analyses. Complex samples procedures were used to accommodate the nested sample design of the ABCD study data. Descriptive statistics summarized demographics of remaining participants (N = 8608, Table 1). The relationship between baseline ELS scores and time to first use of alcohol, nicotine, and marijuana was determined via survival analysis utilizing Cox regression with the assumption of proportional hazards. Hazard ratios were reported after adjustment for covariates of age, puberty, sex (where applicable), and race/ethnicity at baseline. Relationships among substances, dimensions of ELS, and sex were analyzed first in a pooled and then separate manner (i.e., Model 1 analyzed ELS Total, and Model 2 split ELS into individual dimensions of Environmental, Family, and Trauma for analysis). Significance level was set at p < 0.05, with adjusted hazard ratio (aHR) and 95 % confidence intervals reported for each regression model. The primary sampling unit was defined as the site (N = 22) and sample weights were applied using the American Community Survey raked propensity scores provided in the ABCD database. Twins and triplets were removed from the analysis (N = 1860). Frequency analysis provided the N and % of each race/ethnicity with chi-squared comparison. Pubertal development and ELS score means were compared between and within sex and race/ethnicity via T-test or ANOVA respectively, with significance set at p < 0.05. Average age in years of first use of each substance were compared between sexes and presented as the mean and standard error.

## 3. Results

To understand how ELS impacts subsequent substance use in this national diverse sample of youth aged 9–10 at baseline, this study had the following aims: 1) Test the overall associations between ELS and subsequent first substance use, 2) Test these associations for within-sex differences, 3) Test for variation of these effects by ELS type, 4) Explore these associations by substance type.

### 3.1. Descriptive statistics

As shown in Table 1, the average age in months at baseline was 119.14 ± 0.23 months for males and 118.68 ± 0.24 months for females. Baseline parental rating of PDS was significantly higher for females (1.80 ± 0.03) compared to males (1.47 ± 0.02, p < 0.001). Of the dimensional ELS categories, only ELS Family score significantly differed between sexes with males scoring higher (4.80 ± 0.17) than females (4.60 ± 0.16, p = 0.019). There were no significant differences between sexes in age of first use across any substance measured (p > 0.05).

### 3.2. Cox regressions

#### 3.2.1. All substances

**Fig. 1.**
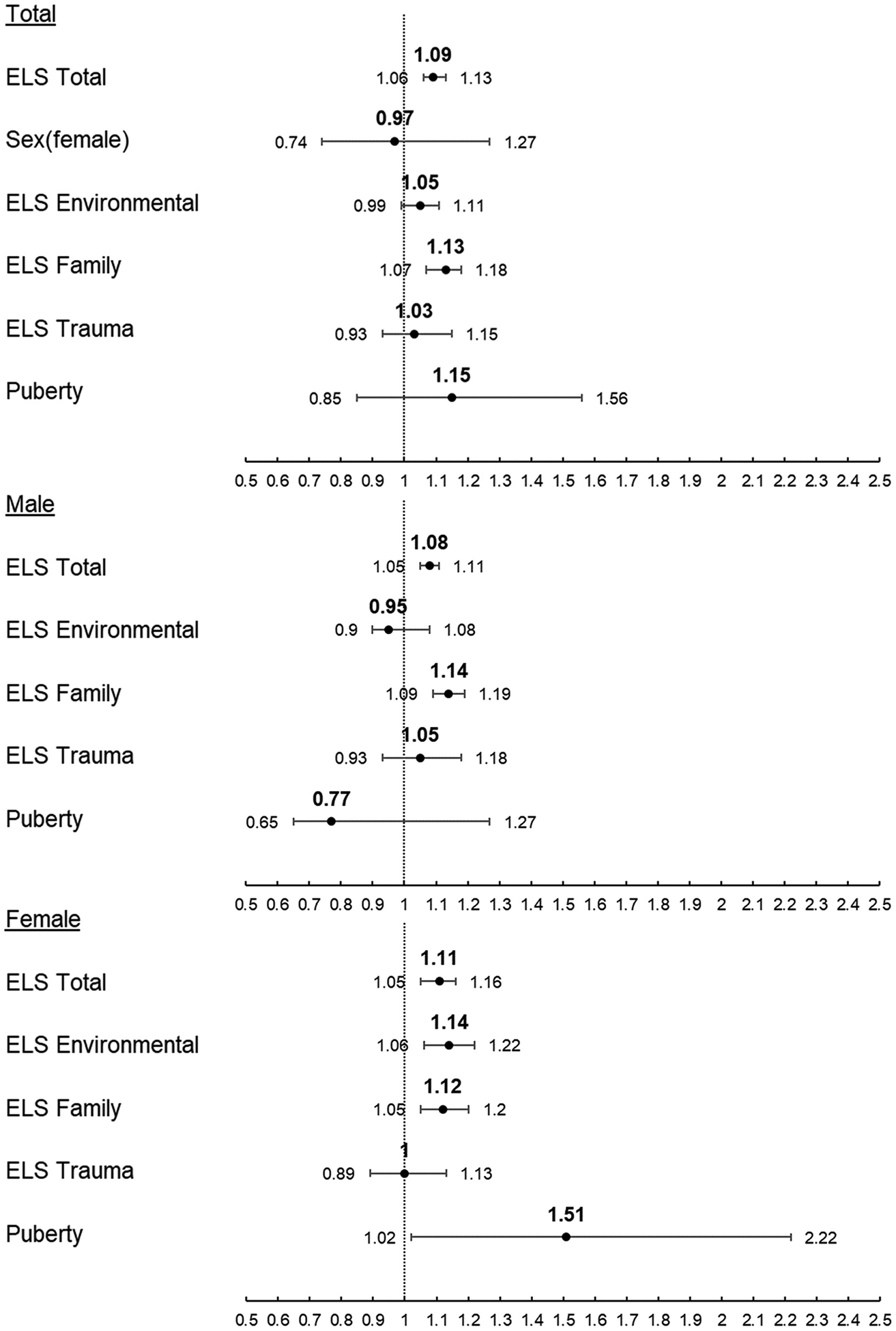
Adjusted Hazard Ratio and 95 % Confidence Interval of Risk of First Use of All Substances Combined. Top: Combined sex sample Middle: Male-only sample Bottom: Female-only sample.

In the initial model (Model 1) of all substances (Fig. 1), ELS Total score was significantly associated with a higher adjusted hazard ratio (aHR, Table 2) of first use of any substance (either alcohol, nicotine, or marijuana). For each 1 unit increase in ELS Total score the rate of first use of any substance increased by 8 % (p < 0.001) for males and 11 % (p < 0.001) for females, however sex was not associated with a higher rate of first use of any substance in the combined-sex sample (p = 0.801). Dimensional analysis of all substances (Model 2) revealed 1 unit increases in either ELS Environmental or ELS Trauma scores did not significantly increase the rate of first use of any substance in the combined-sex sample. However, a 1 unit increase in ELS Family score increased the rate of first use of any substance by 13 % (p < 0.001) in the combined-sex sample (Table 2). Once separated by sex, a 1 unit increase in ELS Family score increased the rate of first use of any substance by 14 % (p < 0.001) for males and 12 % (p = 0.002) for females. In females, a 1 unit increase in ELS Environmental score increased the rate of first use of any substance by 14 % (p < 0.001), while a 1 unit increase in PDS increased the rate of first use of any substance by 51 % (p = 0.040) (Table 2).

In comparison to White individuals in the combined-sex sample, identifying as Asian decreased the rate of first use of any substance across both models (aHR = 0.19, p = 0.035 for Model 1, aHR = 0.20, p = 0.037 for Model 2, Table 2). Females identifying as either Black or Other also decreased the rate of first use of any substance compared to White females across both models (Black aHR = 0.45, p = 0.035, Other aHR = 0.49, p = 0.021 for Model 1, Black aHR = 0.43, p = 0.028, Other aHR = 0.47, p = 0.019 for Model 2). Finally, a 1 month increase in age at baseline increased the rate of first use of any substance by 5 % (p = 0.001) for males and 4 % (p < 0.001) for females across both models.

#### 3.2.2. Alcohol

**Fig. 2.**
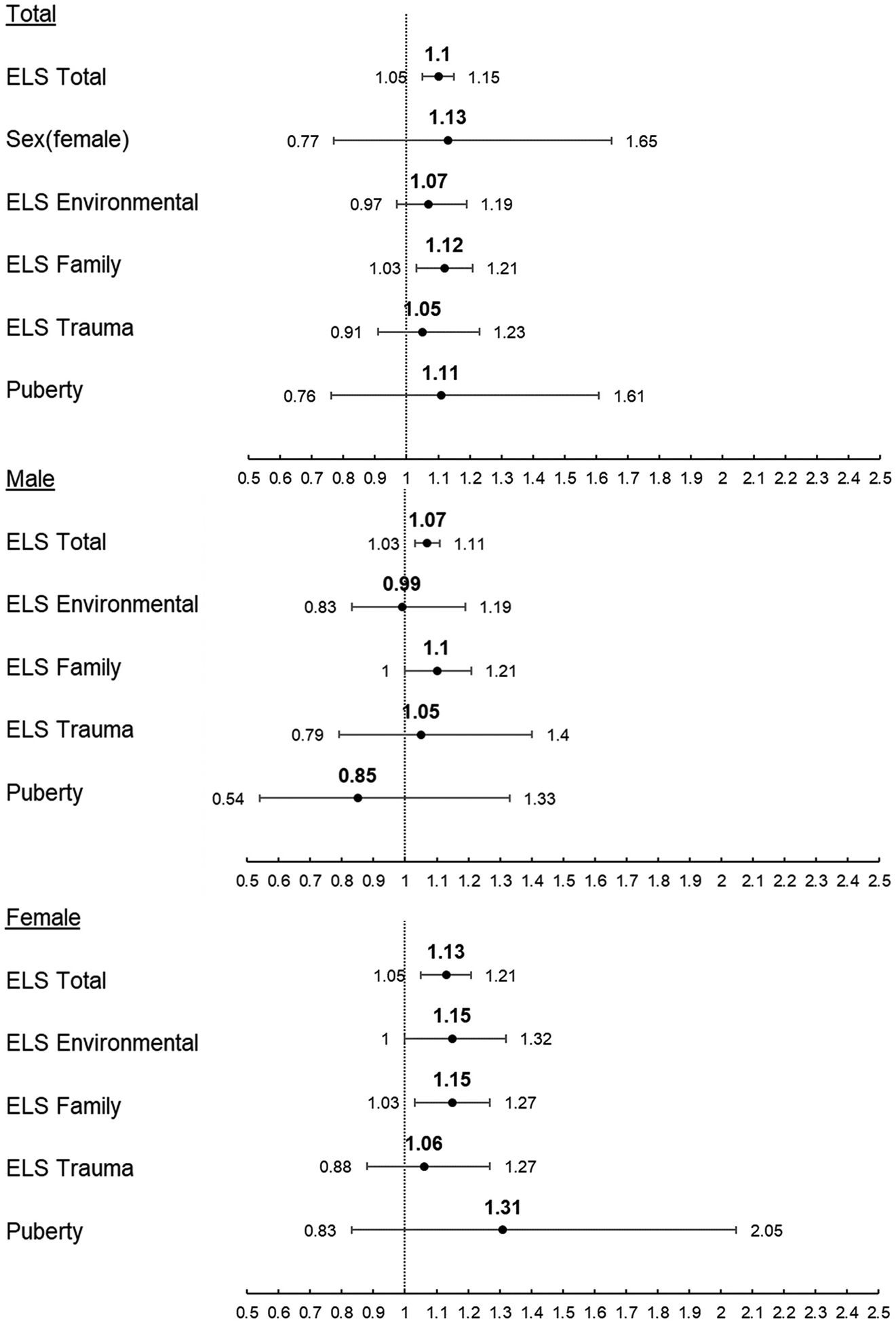
Adjusted Hazard Ratio and 95 % Confidence Interval of Risk of First Use of Alcohol. Top: Combined sex sample Middle: Male-only sample Bottom: Female-only sample.

Model 1 of combined sexes showed a 1 unit increase in ELS Total score increased the rate of first use of alcohol by 10 % (p < 0.001) (Fig. 2). Separating by sex revealed a 1 unit increase in ELS Total score increased the rate of first use of alcohol by 7 % (p = 0.002) for males and 13 % (p = 0.002) for females (Table 3). Dimensional ELS analysis (Model 2) of the combined-sex sample found a 1 unit increase in ELS Family score, but not ELS Environmental or Trauma scores, increased the rate of first use of alcohol by 12 % (p = 0.011). Once separated by sex, a 1 unit increase in either ELS Environmental score (p < 0.050) or ELS Family score (p = 0.011) increased the rate of first use of alcohol by 15 % for females (Table 3). Increase in PDS did not significantly increase or decrease the rate of first use of alcohol in either model for either sex. Within the combined-sex sample of Model 1, identifying as Black (aHR = 0.33, p = 0.024, Table 3) or Other (aHR = 0.32, p = 0.012, Table 3) was associated with a decreased rate of first use of alcohol compared to identifying as White, an effect driven by females (Black aHR = 0.18, p = 0.035, Other aHR = 0.18, p = 0.038). These effects were also observed in Model 2. A 1 month increase in age at baseline increased the rate of first use of alcohol by 7 % (p < 0.001) for males and 4 % (p = 0.034) for females across both models.

#### 3.2.3. Nicotine

**Fig. 3.**
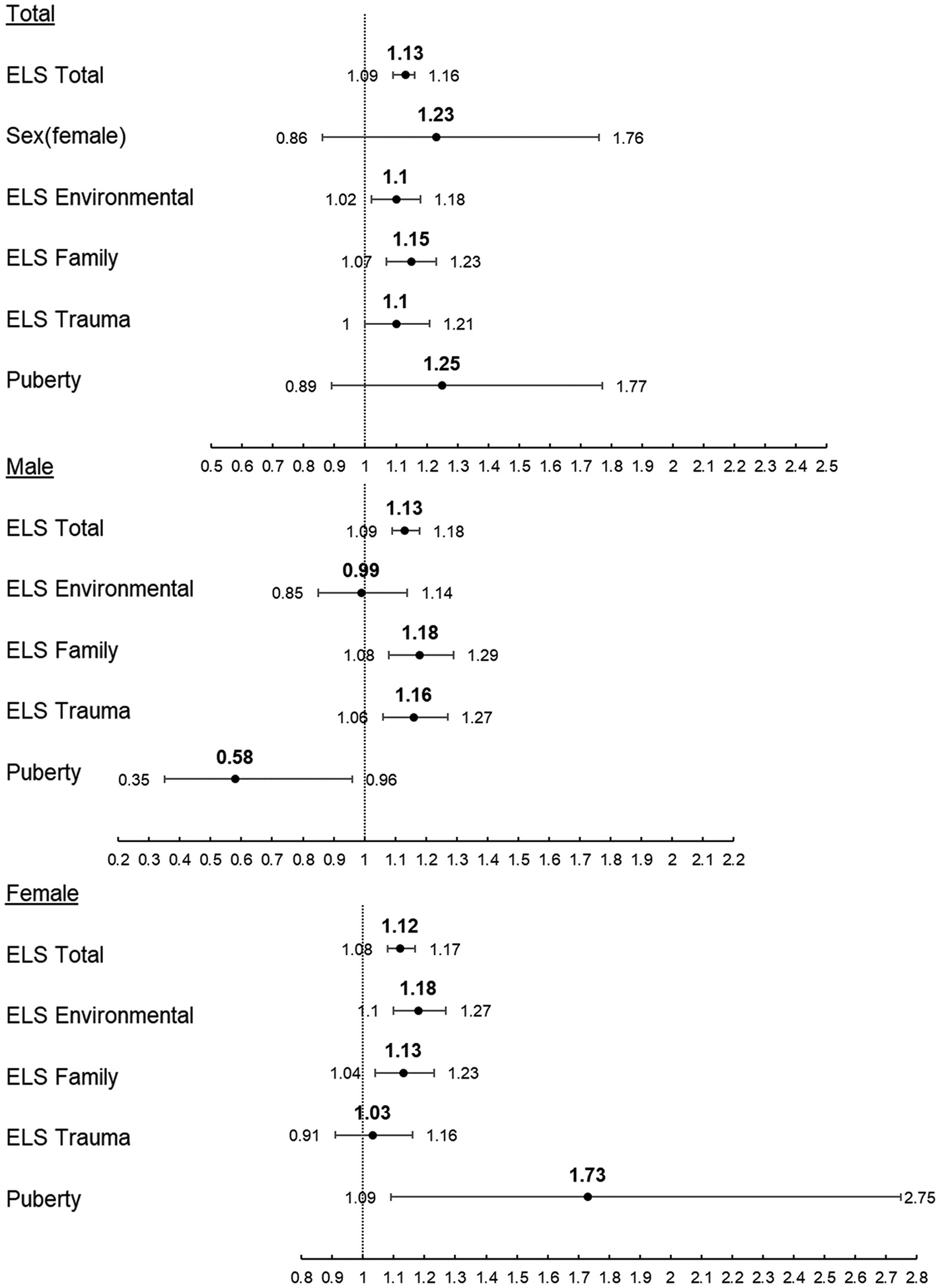
Adjusted Hazard Ratio and 95 % Confidence Interval of Risk of First Use of Nicotine. Top: Combined sex sample Middle: Male-only sample Bottom: Female-only sample.

In the Model 1 analysis of nicotine use among combined sexes, a 1 unit increase in ELS Total score increased the rate of first use of nicotine by 13 % (p < 0.001) (Fig. 3). When separated by sex, a 1 unit increase in ELS Total score increased the rate of first use of nicotine by 13 % (p < 0.001) for males and 12 % (p < 0.001) for females (Table 4). A 1 unit increase in PDS decreased the rate of first use of nicotine by 45 % (p = 0.030) for males and increased the rate of first use of nicotine by 71 % (p = 0.036) for females. Dimensional ELS analysis (Model 2) revealed 1 unit increases in ELS Environmental, ELS Family, or ELS Trauma scores (Fig. 3) increased the rate of first use of nicotine in the combined-sex sample by 10 % (p = 0.018), 15 % (p < 0.001), and 10 % (p = 0.039), respectively. When separating by sex, a 1 unit increase in ELS Environmental score increased the rate of first use of nicotine by 18 % (p < 0.001) for females only. A 1 unit increase in ELS Family score increased the rate of first use of nicotine by 18 % (p = 0.001) for males and 13 % (p = 0.004) for females. Finally, a 1 unit increase in ELS Trauma score increased the rate of first use of nicotine by 16 % (p = 0.002) for males but not females (p = 0.615) (Table 4). In Model 2, each 1 unit increase in PDS decreased the rate of first use of nicotine by 42 % (p = 0.036) for males and increased the rate of first use of nicotine by 73 % (p = 0.023) for females (Table 4). Compared to identifying as White, identifying as Black decreased the rate of first use of nicotine (aHR = 0.60, p = 0.022) across both models in the combined-sex sample, an effect which was driven by females (aHR = 0.44, p = 0.017 Model 1, aHR = 0.40, p = 0.013 Model 2). Additionally, females identifying as Other (compared to White) decreased the rate of first use of nicotine (aHR = 0.32, p = 0.014, Model 1, aHR = 0.31, p = 0.005 Model 2, Table 4) in both models. A 1 month increase in age at baseline increased the rate of first use of nicotine by 5 % (p < 0.001) in the combined-sex sample; separating by sex showed the effect was driven by females (aHR = 1.06, p < 0.001, Table 4) across both models.

#### 3.2.4. Marijuana

**Fig. 4.**
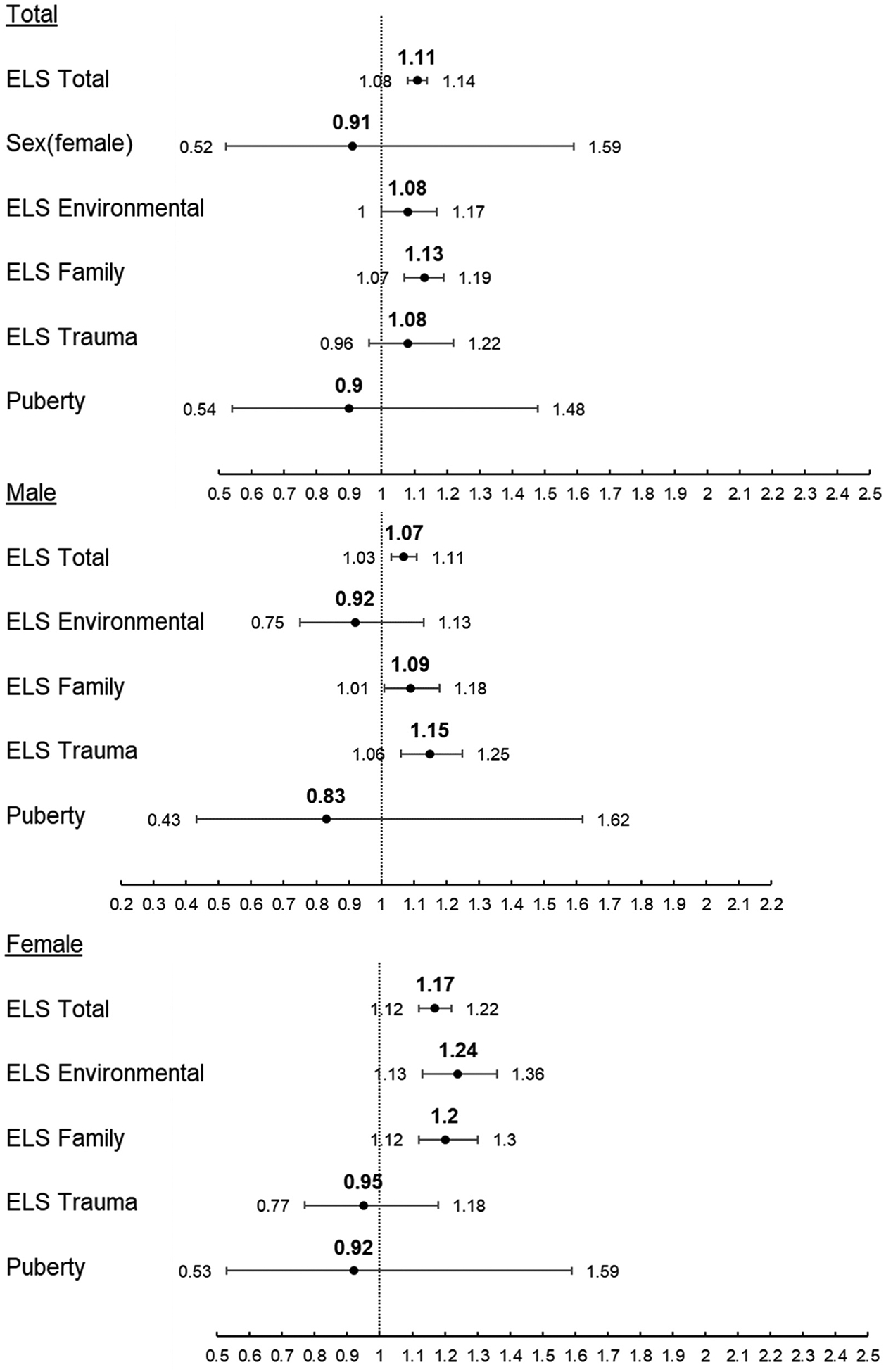
Adjusted Hazard Ratio and 95 % Confidence Interval of Risk of First Use of Marijuana. Top: Combined sex sample Middle: Male-only sample Bottom: Female-only sample.

In Model 1, a 1 unit increase in ELS Total score increased the rate of first use of marijuana by 11 % (p < 0.001) in the combined-sex sample. Separating by sex, a 1 unit increase in ELS Total score increased the rate of first use of marijuana by 7 % (p = 0.001) in males and 17 % (p < 0.001) in females (Fig. 4). Dimensional ELS analysis of the combined-sex sample revealed a 1 unit increase in ELS Family score increased the rate of first use of marijuana by 13 % (p < 0.001); separating by sex revealed a 9 % (p = 0.026) increase in the rate of first use of marijuana for males and a 20 % (p < 0.001) increase in females for each 1 unit increase in ELS Family score. Other dimensions of ELS varied in their effects by sex (Table 5). A 1 unit increase in ELS Environmental score increased the rate of first use of marijuana by 24 % (p < 0.001) for females, whereas a 1 unit increase in ELS Trauma score increased the rate of first use of marijuana by 15 % (p = 0.003) for males. A 1 unit increase in PDS did not alter the rate of first use of marijuana in either model (p > 0.05) (Table 5). In Model 2, identifying as a Hispanic male rather than a White male increased the rate of first use of marijuana (aHR = 2.06, p = 0.040, Table 5). A 1 month increase in age at baseline increased the rate of first use of marijuana by 5 % (p = 0.009) in males and 6 % (p = 0.006) in females.

## 4. Discussion

### 4.1. ELS and adolescent substance use

The present study assessed combined and disaggregated risk factors (ELS scores) for use of multiple substances within and between sexes in the ABCD population. Results of combined-sex analyses show that higher ELS scores are associated with an increase in risk of first use of alcohol (10 %), nicotine (13 %), or marijuana (11 %). The cumulative ELS Total score captures a small but statistically significant effect present among the broad population of adolescents in the ABCD Study. Other studies identifying links between ELS and substance use rely on retrospective measures from specific higher-risk subpopulations (Aschengrau et al., 2023; Evans et al., 2017; Rothman et al., 2008; Zaidi et al., 2022), limiting the applicability of findings. Associations in the current study suggest ELS is a risk factor for substance use among all adolescents and even low-level ELS can increase the risk of substance use by age 13.

Splitting ELS Total into separate dimensions of Environmental, Family, and Trauma revealed substance-specific effects. ELS Environmental scores were associated with a 10 % higher risk of nicotine use, matching the trend seen in the 2022 Monitoring the Future study where parental education (used in the study as a proxy for SES) held an inverse relationship with lifetime prevalence of cigarette use and e-cigarette use among 8th graders (Miech et al., 2023). Like ELS Environmental scores, ELS Trauma scores were only associated with nicotine use, where individuals with higher Trauma scores were at 10 % higher risk of first nicotine use. Traumatic events have been associated with later substance use in adolescence, including nicotine (Patel et al., 2024). This association may represent the development of nicotine use as a coping mechanism for depression and anxiety symptoms occurring because of trauma (Patel et al., 2024). Links between symptoms and nicotine use have been found cross-sectionally (Klein et al., 2022) and longitudinally among adolescents, with more evidence for symptomology precipitating nicotine use than vice versa (Lee et al., 2023).

It is important to note that nicotine has the highest prevalence of initiation within the study. Although adolescent cigarette use has continued to decline in recent years, the incidence of e-cigarette use has remained high. National trends show an incidence of 15.2 % among 8th graders for any e-cigarette use, compared to an incidence of 5 % for cigarette use (Miech et al., 2023). Thus, the significance of ELS Environmental and Trauma scores as predictors of nicotine use may be driven by the increased prevalence of nicotine initiation among adolescents. As the ABCD study cohort ages, the prevalence of alcohol or marijuana use initiation will likely increase, and ELS environmental and Trauma scores may prove to be significant predictors.

### 4.2. ELS and sex differences

Though our study found no main effect of sex in the combined analyses, performing single-sex analyses resulted in significant differences within sexes across ELS dimensions and substances. ELS Total scores were consistently associated with higher risk of any substance use in both male and female samples, with females showing larger associations with risk than males for all substances except nicotine. This suggests that the factors comprising ELS Total scores are impacting risk of first substance use more strongly in females than in males, though no direct comparisons between male and female hazard ratios were made. The existence of a sex or gender difference between the effects of ELS on later substance use is debated in the literature, but adolescent and adult studies have shown that females with higher levels of ELS are more likely than males to use multiple substances (Evans et al., 2017; Zaidi et al., 2022).

Analysis by sex revealed ELS Environment scores to be associated with a 14–24 % higher risk of first use of all three substances in females but not males. This contrasts with previous work by Fagan et al. (2015) which found no direct effects of neighborhood stressors on adolescent substance use, though the authors of the study noted they did not look at indirect effects. The sex-specific findings of the current study suggest the higher risk of first use associated with ELS Environment scores may be due to the relationship between SES and early puberty among females. The mechanism by which puberty mediates the relationship between ELS and substance use is still under investigation, but these results fit with previous studies linking lower SES with earlier puberty in females (Ding et al., 2024; Kelly et al., 2017). Other studies have also found an association between household status (divorced, widowed, separated) and early puberty in females (Aghaee et al., 2020; DiLalla et al., 2021) which may contribute to the significance of ELS Environment scores among females but not males. Early puberty leads to association with older substance-using peers (Kuhn, 2015) and increased internalizing symptomology (Niu et al., 2023), for which substance use becomes a coping mechanism (Niu et al., 2023; Romano et al., 2021). Though pubertal development at baseline was associated with a 73 % higher risk of first nicotine use for females and a 42 % lower risk for males, this measure of puberty was not assessed with respect to the relative timing of development among peers, and thus no conclusions regarding “early” or “late” pubertal timing can be derived from the current study. Additionally, ELS Environment scores continued to be significantly associated with higher risk of use initiation after incorporating puberty into the model, suggesting effects beyond those associated with puberty.

Combined-sex analysis showed ELS Family scores were associated with higher risk of first use of all substances by 12–15 %. The wide-spread effect of ELS Family is not surprising, given that parental psychopathology and family conflict are often identified as risk factors for adolescent substance use (Nawi et al., 2021; Wade et al., 2022). Additionally, the presence of a warm supportive family environment is critical for the development of resilience which buffers existing predisposing factors for adolescent substance use (Aschengrau et al., 2023; Pugh and Richter, 2023). Yet, analysis by sex showed sex- and substance-specific effects. Males did not show an association with any ELS dimension scores (Environmental, Family, or Trauma) and risk of first alcohol use, despite the observed association between ELS Total score and risk of first alcohol use. This suggests a less direct relationship between ELS and male alcohol use involving other factors not measured here such as prenatal stressors. In comparison, female ELS Family scores were associated with a 15 % higher risk of first alcohol use. While ELS Family score associations were similar across males and females for nicotine (18 % higher risk for males, 13 % higher risk for females), results for marijuana showed the association was larger among females (20 % higher risk) than for males (9 % higher risk). Overall, these sex differences indicate that although parental warmth and support have been found to be equally protective against substance use between males and females (Pugh and Richter, 2023), studies should examine sex-specific effects when assessing family-related risk factors for substance use.

The significant association between higher ELS Trauma scores and higher risk of first nicotine (16 %) and marijuana (15 %) use among males is surprising, given that there were no effects of ELS Trauma present in females. This suggests there may be other factors at play in the male sample that are not captured here, potentially peer influences (Martz et al., 2022) or development of mental health disorders (Sullivan et al., 2022) which may increase substance use. For example, a recent study found that ELS increased male delinquency, and socializing with other delinquent peers was associated with increased odds of substance use initiation (Gajos et al., 2023). Therefore, the significance of the association between ELS Trauma and the risk of first nicotine or marijuana use in males may be through increased rule-breaking, delinquency, and affiliating with delinquent peers.

### 4.3. Age and race/ethnicity

Age at baseline was associated with higher risk of first use across models, sexes, and substances. This matches the literature, as an increase in substance use from ages 11–16 has been observed across multiple decades from 1975 to 2022 (Johnston et al., 2022), and age at baseline is positively associated with substance familiarity (Bhatia et al., 2023), which increases odds of future use. Effects of race/ethnicity on risk of first use were substance- and sex-specific. Identifying as Asian was a protective factor when compared to White individuals, lowering the risk of first use of any substance. In fact, the number of Asian females reporting use of nicotine or marijuana was insufficient for reliable analyses, and participants identifying as Asian represented 2 % of the population in the current study. Identifying as Black or Other was protective among females, as these identities were associated with a lower risk of first use of alcohol and nicotine compared to White females. This is consistent with the literature (Amaro et al., 2001) and likely an effect of strong resilience factors, such as ethnic identity, social support, and psychological empowerment (Caldwell et al., 2002), which have been associated with a decrease in substance use among Black girls (Opara et al., 2022). Surprisingly, Hispanic males showed a 206 % higher risk of first marijuana use, though the range of this effect is quite large (see Table 5 for 95 % CI). This higher risk may be due to differences in substance availability, as another study utilizing ABCD data found Hispanic parents were most likely to report easy availability of marijuana when compared to other race/ethnicities (Martz et al., 2022). Overall, the effects of race/ethnicity remained after adjusting for ELS Environmental scores, indicating these contrasting effects come from cultural or social sources rather than SES and neighborhood deprivation factors.

### 4.4. Limitations and future directions

The current study aimed to establish a connection between ELS and substance use that may later be tested for mediation by salivary hormones and moderation by sex (Donovan et al., 2024; Koss and Gunnar, 2018). ELS scores and covariates were assessed cross-sectionally at baseline, and it is possible that longitudinal changes in these measures could have influenced time to first substance use within the study. Future studies may incorporate ELS and pubertal development assessments across multiple time points as time-varying predictors or pubertal timing may act as a mediator in the link between ELS and substance use. Mediation effects of baseline pubertal development score were not explored in the current study. Future analysis assessing mediating effects of pubertal timing and tempo on ELS score and later substance use is warranted.

The definition of ELS utilized here does not include pre-natal exposures, genetic polymorphisms and epigenetic markers, social influences (e.g., peer use, parental rules, substance availability) or individual factors such as psychopathology and personality traits, as other studies have already performed these analyses (Bhatia et al., 2023; Green et al., 2024; Martz et al., 2022; Sullivan et al., 2022; Wade et al., 2021). However, previous analyses did not examine these factors in models separating by sex or substance, and few incorporated any pubertal factors. Future studies should consider the role of sex and puberty in their examination of social and individual factors contributing to alcohol, nicotine, or marijuana use initiation.

Finally, at the data collection wave included in this study participants were still in middle school, and previous studies indicate that adolescent substance use peaks during high school. Therefore, the full impact of ELS influences on substance use may not yet be captured with the current analyses. Incorporation of future waves of data will expand the sample size and allow for further investigation of sex differences in the link between ELS and risk of first substance use.

## 5. Conclusion

The current study identified factors that increase the risk of substance use initiation among ELS-exposed adolescents and assessed sex differences in their impact. The likelihood of substance use depends on sex, type of ELS experienced, and the substance in question. The persistent significance of the ELS Family score on time to substance use across analyses suggests targeting family function and cohesion may prove most effective. In fact, early and middle childhood interventions such as the Family Check Up have shown reductions in adolescent psychopathology and substance use, helping to ameliorate the negative effects of growing up in a low-income household (Tein et al., 2023). Improving family support and educating parents on the prevalence of early adolescent substance use have also been effective in reducing adolescent substance use and improving family cohesion (Botzet et al., 2019). While policies targeting family-based and community interventions (Aytur et al., 2022; Nair et al., 2022) address the most malleable dimension of ELS (Family), addressing the Environmental and Trauma dimensions of ELS requires larger scale changes involving social determinants of health. These types of change require large bodies of evidence to which the current study contributes. Finally, as evidenced by our analysis; aggregate measures obscure important relationships that may help identify sex differences in the link from ELS to substance use initiation. The current study highlights the importance of investigating differences within and between sexes by utilizing sex and gender to discover the nuances of ELS and its effect on adolescent substance use.

## Supplementary Material

Supplemental material

## Figures and Tables

**Table 1:** Demographic information of included study population and separated by sex. Age in months, race/ethnicity, Pubertal Development Score, dimensional ELS scores, Total ELS scores by race/ethnicity, average age in years of first use by substance and sex.

Population demographics	Subjects All	Male	Female	p value
Age 9–10 years	(N = 8608)	(N = 4533, 52.66 %)	(N = 4075, 47.34 %)	(*t*-test, m vs f)
Age in months (mean, SE)	118.92 (0.21)	119.14 (0.23)	118.68 (0.24)	***0.041***
Race/Ethnicity (N, % of total)				0.659
Asian	194 (2.25 %)	97 (2.14 %)	97 (2.38 %)	
Black	1220 (14.17 %)	623 (13.74 %)	597 (14.65 %)	
Hispanic	1815 (21.09 %)	943 (20.80 %)	872 (21.40 %)	
Other	935 (10.86 %)	481 (10.61 %)	454 (11.14 %)	
White	4444 (51.63 %)	2389 (52.70 %)	2055 (50.43 %)	
Pubertal Development Score (mean, SE)	1.63 (0.02)	1.47 (0.02)	1.80 (0.03)	**< *0.001***
**ELS scores by type (mean, SE)**				
Environmental (0–11)	1.14 (0.10)	1.14 (0.10)	1.14 (0.11)	0.928
Family (0–22)	4.70 (0.16)	4.80 (0.17)	4.60 (0.16)	***0.019***
Trauma (0–17)	0.56 (0.03)	0.55 (0.04)	0.57 (0.03)	0.557
Total (0–50)	6.40 (0.21)	6.48 (0.23)	6.31 (0.24)	0.079
**ELS Total by race/ethnicity (mean, SE)**				
Asian	3.55 (0.22)	3.37 (0.27)	3.74 (0.31)	0.353
Black	7.60 (0.31)	7.78 (0.23)	7.42 (0.41)	0.135
Hispanic	6.46 (0.37)	6.51 (0.41)	6.40 (0.34)	0.419
Other	7.52 (0.36)	7.56 (0.35)	7.47 (0.42)	0.756
White	6.13 (0.22)	6.24 (0.23)	6.00 (0.22)	***0.027***
**Average age in years of first use by substance (mean, SE)**				
Any	12.27 (0.07)	12.22 (0.08)	12.33 (0.10)	0.269
Alcohol	12.64 (0.09)	12.67 (0.09)	12.62 (0.18)	0.814
Nicotine	12.47 (0.08)	12.36 (0.15)	12.58 (0.08)	0.222
Marijuana	12.05 (0.12)	11.96 (0.11)	12.17 (0.19)	0.275

**Table 2:** Adjusted Hazard Ratio and 95 % CI for All Substances.

Total ELS (Model 1) All Substances									
Total Sample (n = 8608)				Male Sample (n = 4533)			Female Sample (n = 4057)		
	Adjusted Hazard Ratio	95 %CI	p value	Adjusted Hazard Ratio	95 %CI	p value	Adjusted Hazard Ratio	95 %CI	p value
**ELS Total**	1.09	1.06–1.13	**< *0.001***	1.08	1.05–1.11	**< *0.001***	1.11	1.05–1.16	**< *0.001***
**Sex (female)**	0.97	0.74–1.27	0.801						
**Puberty**	1.13	0.83–1.55	0.406	0.73	0.46–1.18	0.191	1.49	0.99–2.24	0.056
**Race:**			***0.061***			0.526			***0.019***
**Asian**	0.19	0.04–0.88	***0.035***	0.41	0.10–1.17	0.209	n/a	n/a	n/a
**Black**	0.73	0.47–1.12	0.141	1.13	0.69–1.85	0.605	0.45	0.22–0.94	***0.035***
**Hispanic**	1.10	0.76–1.60	0.594	1.26	0.83–1.93	0.265	0.98	0.60–1.59	0.926
**Other**	0.83	0.50–1.38	0.454	1.22	0.57–2.61	0.592	0.49	0.27–0.89	***0.021***
**White**	Reference			Reference			Reference		
**Age (9–10 y)**	1.05	1.03–1.07	**< *0.001***	1.05	1.03–1.07	**< *0.001***	1.04	1.02–1.07	***0.001***
**Dimensional ELS (Model 2) All Substances**									
**ELS Environmental**	1.05	0.99–1.11	0.107	0.95	0.90–1.08	0.329	1.14	1.06–1.22	***0.001***
**ELS Family**	1.13	1.07–1.18	**< *0.001***	1.14	1.09–1.19	**< *0.001***	1.12	1.05–1.20	***0.002***
**ELS Trauma**	1.03	0.93–1.15	0.519	1.05	0.93–1.18	0.425	1.00	0.89–1.13	0.941
**Sex (female)**	0.97	0.74–1.26	0.813						
**Puberty**	1.15	0.85–1.56	0.334	0.77	0.65–1.27	0.225	1.51	1.02–2.22	***0.040***
**Race:**			0.062			0.343			***0.016***
**Asian**	0.20	0.04–0.90	***0.037***	0.43	0.10–1.78	0.231	n/a	n/a	n/a
**Black**	0.77	0.49–1.20	0.230	1.32	0.80–2.20	0.260	0.43	0.20–0.90	***0.028***
**Hispanic**	1.15	0.78–1.72	0.460	1.44	0.91–2.30	0.114	0.94	0.57–1.56	0.806
**Other**	0.85	0.51–1.41	0.508	1.29	0.59–2.86	0.504	0.47	0.26–0.87	***0.019***
**White**	Reference			Reference			Reference		
**Age (9–10 y)**	1.05	1.03–1.07	**< *0.001***	1.05	1.04–1.07	**< *0.001***	1.04	1.02–1.07	***0.001***

Note in both models, sample size of Asian females was too low to generate a reliable estimate. Bold, italicized p values indicate significance at p < 0.05.

**Table 3:** Adjusted Hazard Ratio and 95 % CI for Alcohol.

Total ELS (Model 1) Alcohol									
Total Sample (n = 8608)				Male Sample (n = 4533)			Female Sample (n = 4057)		
	Adjusted Hazard Ratio	95 %CI	p value	Adjusted Hazard Ratio	95 %CI	p value	Adjusted Hazard Ratio	95 %CI	p value
**ELS Total**	1.10	1.05–1.15	**< *0.001***	1.07	1.03–1.11	***0.002***	1.13	1.05–1.21	***0.002***
**Sex (female)**	1.13	0.77–1.65	0.513						
**Puberty**	1.10	0.75–1.61	0.623	0.82	0.53–1.29	0.381	1.29	0.81–2.04	0.269
**Race:**			***0.035***			0.491			0.053
**Asian**	n/a	n/a	n/a	n/a	n/a	n/a	n/a	n/a	n/a
**Black**	0.33	0.13–0.85	***0.024***	0.56	0.17–1.82	0.320	0.18	0.04–0.87	***0.035***
**Hispanic**	0.77	0.46–1.28	0.294	0.79	0.37–1.71	0.539	0.75	0.45–1.24	0.248
**Other**	0.32	0.14–0.76	***0.012***	0.51	0.19–1.38	0.174	0.18	0.03–0.90	***0.038***
**White**	Reference			Reference			Reference		
**Age (9–10 y)**	1.06	1.03–1.09	***0.001***	1.07	1.04–1.11	**< *0.001***	1.05	1.00–1.10	***0.035***
**Dimensional ELS (Model 2) Alcohol**									
**ELS Environmental**	1.07	0.97–1.19	0.163	0.99	0.83–1.19	0.965	1.15	1.00–1.32	**< *0.050***
**ELS Family**	1.12	1.03–1.21	***0.011***	1.10	1.00–1.21	0.056	1.15	1.03–1.27	***0.011***
**ELS Trauma**	1.05	0.91–1.23	0.470	1.05	0.79–1.40	0.734	1.06	0.88–1.27	0.526
**Sex (female)**	1.13	0.78–1.64	0.508						
**Puberty**	1.11	0.76–1.61	0.568	0.85	0.54–1.33	0.450	1.31	0.83–2.05	0.234
**Race:**			***0.042***			0.520			0.065
**Asian**	n/a	n/a	n/a	n/a	n/a	n/a	n/a	n/a	n/a
**Black**	0.34	0.13–0.93	***0.036***	0.612	0.19–2.00	0.398	0.18	0.04–0.84	***0.031***
**Hispanic**	0.78	0.44–1.39	0.384	0.852	0.35–2.05	0.707	0.73	0.44–1.21	0.211
**Other**	0.33	0.14–0.78	***0.015***	0.52	0.19–1.45	0.199	0.17	0.03–0.95	***0.044***
**White**	Reference			Reference			Reference		
**Age (9–10 y)**	1.06	1.03–1.09	***0.001***	1.07	1.04–1.11	**< *0.001***	1.05	1.00–1.10	***0.034***

Note in both models, sample size of Asian females was too low to generate a reliable estimate. Bold, italicized p values indicate significance at p < 0.05.

**Table 4:** Adjusted Hazard Ratio and 95 % CI for Nicotine.

Total ELS (Model 1) Nicotine									
Total Sample (n = 8608)				Male Sample (n = 4533)			Female Sample (n = 4057)		
	Adjusted Hazard Ratio	95 %CI	p value	Adjusted Hazard Ratio	95 %CI	p value	Adjusted Hazard Ratio	95 %CI	p value
**ELS Total**	1.13	1.09–1.16	**< *0.001***	1.13	1.09–1.18	**< *0.001***	1.12	1.08–1.17	**< *0.001***
**Sex (female)**	1.23	0.86–1.76	0.251						
**Puberty**	1.24	1.01–1.52	0.237	0.55	0.33–0.94	***0.030***	1.71	1.04–2.83	***0.036***
**Race:**			0.057			0.573			***0.009***
**Asian**	0.23	0.03–1.89	0.162	0.61	0.07–5.01	0.628	n/a	n/a	n/a
**Black**	0.60	0.39–0.92	***0.022***	0.93	0.46–1.87	0.823	0.44	0.23–0.85	***0.017***
**Hispanic**	1.33	0.80–2.19	0.255	1.48	0.73–3.01	0.261	1.24	0.66–2.32	0.482
**Other**	0.85	0.42–1.71	0.636	1.68	0.67–4.25	0.255	0.32	0.14–0.78	***0.014***
**White**	Reference			Reference			Reference		
**Age (9–10 y)**	1.05	1.03–1.07	**< *0.001***	1.04	1.00–1.08	0.074	1.06	1.03–1.08	**< *0.001***
**Dimensional ELS (Model 2) Nicotine**									
**ELS Environmental**	1.10	1.02–1.18	***0.018***	0.99	0.85–1.14	0.856	1.18	1.10–1.27	**< *0.001***
**ELS Family**	1.15	1.07–1.23	**< *0.001***	1.18	1.08–1.29	***0.001***	1.13	1.04–1.23	***0.004***
**ELS Trauma**	1.10	1.00–1.21	***0.039***	1.16	1.06–1.27	***0.002***	1.03	0.91–1.16	0.615
**Sex (female)**	1.23	0.86–1.76	0.248						
**Puberty**	1.25	0.89–1.77	0.182	0.58	0.35–0.96	***0.036***	1.73	1.09–2.75	***0.023***
**Race:**			0.066			0.491			***0.003***
**Asian**	0.24	0.03–1.91	0.166	0.63	0.08–5.08	0.648	n/a	n/a	n/a
**Black**	0.62	0.39–0.99	***0.046***	1.10	0.51–2.37	0.797	0.40	0.20–0.81	***0.013***
**Hispanic**	1.36	0.80–2.32	0.237	1.71	0.83–3.53	0.141	1.16	0.59–2.27	0.648
**Other**	0.86	0.43–1.72	0.663	1.83	0.70–4.75	0.203	0.31	0.14–0.67	***0.005***
**White**	Reference			Reference			Reference		
**Age (9–10 y)**	1.05	1.03–1.07	**< *0.001***	1.04	1.00–1.08	0.076	1.06	1.03–1.09	**< *0.001***

Note in both models sample size of Asian females was too low to generate a reliable estimate. Bold, italicized p values indicate significance at p < 0.05.

**Table 5:** Adjusted Hazard Ratio and 95 % CI for Marijuana.

Total ELS (Model 1) Marijuana									
Total Sample (n = 8608)				Male Sample (n = 4533)			Female Sample (n = 4057)		
	Adjusted Hazard Ratio	95 %CI	p value	Adjusted Hazard Ratio	95 %CI	p value	Adjusted Hazard Ratio	95 %CI	p value
**ELS Total**	1.11	1.08–1.14	**< *0.001***	1.07	1.03–1.11	***0.001***	1.17	1.12–1.22	**< *0.001***
**Sex (female)**	0.91	0.52–1.59	0.718						
**Puberty**	0.89	0.53–1.47	0.625	0.80	0.41–1.57	0.506	0.88	0.48–1.60	0.666
**Race:**			0.299			0.417			0.537
**Asian**	0.67	0.15–3.06	0.588	1.09	0.24–4.93	0.909	n/a	n/a	n/a
**Black**	1.50	0.82–2.74	0.175	1.86	0.75–4.56	0.167	1.17	0.46–3.00	0.725
**Hispanic**	1.73	0.96–3.12	0.067	1.79	0.94–3.42	0.076	1.65	0.70–3.90	0.242
**Other**	1.28	0.76–2.15	0.343	1.08	0.37–3.15	0.881	1.45	0.61–3.46	0.381
**White**	Reference			Reference			Reference		
**Age (9–10 y)**	1.05	1.02–1.08	***0.001***	1.05	1.01–1.09	***0.010***	1.06	1.02–1.10	***0.006***
**Dimensional ELS (Model 2) Marijuana**									
**ELS Environmental**	1.08	1.00–1.17	0.054	0.92	0.75–1.13	0.425	1.24	1.13–1.36	**< *0.001***
**ELS Family**	1.13	1.07–1.19	**< *0.001***	1.09	1.01–1.18	***0.026***	1.20	1.12–1.30	**< *0.001***
**ELS Trauma**	1.08	0.96–1.22	0.193	1.15	1.06–1.25	***0.003***	0.95	0.77–1.18	0.639
**Sex (female)**	0.91	0.52–1.59	0.721						
**Puberty**	0.90	0.54–1.48	0.656	0.83	0.43–1.62	0.572	0.92	0.53–1.59	0.743
**Race:**			0.250			0.264			0.574
**Asian**	0.68	0.15–3.08	0.601	1.11	0.25–5.00	0.885	n/a	n/a	n/a
**Black**	1.56	0.87–2.77	0.126	2.20	0.90–5.45	0.084	1.05	0.38–2.86	0.927
**Hispanic**	1.78	0.98–3.23	0.057	2.06	1.04–4.08	***0.040***	1.51	0.61–3.76	0.353
**Other**	1.29	0.78–2.14	0.300	1.16	0.39–3.51	0.778	1.38	0.55–3.46	0.471
**White**	Reference			Reference			Reference		
**Age (9–10 y)**	1.05	1.02–1.08	***0.001***	1.05	1.01–1.09	***0.009***	1.06	1.02–1.10	***0.006***

Note in both models, sample size of Asian females was too low to generate a reliable estimate. Bold, italicized p values indicate significance at p < 0.05.
