# Supplementary material for: Early life stress and pubertal predictors of subsequent substance use in a national diverse sample of adolescents: Sex and substance type matter
Source: Drug Alcohol Depend. Author manuscript; Available in PMC 2025 Mar 1. (PMC11849747; doi:10.1016/j.drugalcdep.2025.112551)
Supplement: Supplemental material [file NIHMS2057982-supplement-Supplemental_material.docx]

Supplementary Table 1: Traumatic Events list

| KSADS– PTSD |
| --- |
| 1. A car accident in which your child or another person in the car was hurt bad enough to require medical attention  2. Another significant accident for which your child needed specialized and intensive medical treatment  3. Witnessed or caught in a fire that caused significant property damage or personal injury  4. Witnessed or caught in a natural disaster that caused significant property damage or personal injury  5. Witnessed or present during an act of terrorism (e.g., Boston marathon bombing)  6. Witnessed death or mass destruction in a war zone  7. Witnessed someone shot or stabbed in the community  8. Shot, stabbed, or beaten brutally by a non-family member  9. Shot, stabbed, or beaten brutally by a grown up in the home  10. Beaten to the point of having bruises by a grown up in the home  11. A non-family member threatened to kill your child  12. A family member threatened to kill your child  13. Witness the grownups in the home push, shove or hit one another  14. A grown up in the home touched your child in their privates, had your child touch their privates, or did other sexual things to your child  15. An adult outside your family touched your child in their privates, had your child touch their privates or did other sexual things to your child  16. A peer forced your child to do something sexually  17. Learned about the sudden unexpected death of a loved one |
